# Factors associated with laboratory-confirmed measles cases during the 2023 and 2024 outbreaks in Tanzania—a mixed-methods study

**DOI:** 10.3389/fpubh.2026.1818581

**Published:** 2026-04-29

**Authors:** Fausta Michael, Janeth Peter, Mariam M. Mirambo, Gerald Misinzo, Stephen E. Mshana

**Affiliations:** 1 Immunization and Vaccine Development Program, Ministry of Health, Dodoma, Tanzania; 2 Department of Microbiology and Immunology, Weill Bugando School of Medicine, Catholic University of Health and Allied Sciences, Mwanza, Tanzania; 3 School of Public Health, Catholic University of Health and Allied Sciences, Mwanza, Tanzania; 4 OR Tambo Africa Research Chair for Viral Epidemics, SACIDS Foundation for One Health, Sokoine University of Agriculture, Morogoro, Tanzania

**Keywords:** factors, measles, outbreaks 2023 and 2024, Tanzania, vaccination

## Abstract

**Background:**

Despite the introduction of the two-dose measles-containing vaccine (MCV2) in 2014, measles remains a persistent public health problem in Tanzania, characterized by recurrent outbreaks and shifting epidemiological patterns. Understanding the epidemiology of measles in the country is therefore essential to inform context-specific elimination strategies.

**Methods:**

During the 2023 and 2024 measles outbreaks, data and samples from the National Measles Epidemiological Surveillance System were used. Sera were processed to detect specific Measles Immunoglobulin M (IgM) antibodies using an indirect enzyme-linked immunosorbent assay. In addition, in-depth interviews and focus group discussions were conducted with key stakeholders and caregivers, respectively, to explore factors associated with measles outbreaks.

**Results:**

A total of 17,902 suspected measles cases were enrolled; of these, 11,576 (64.7%) were confirmed measles cases. The overall incidence rate declined markedly from 163.9 cases per 1,000,000 population in 2023 to 15.4 cases per 1,000,000 population in 2024, with the highest incidence occurring among children aged <1 year. Of the total confirmed measles cases, 10,239 (57.2%), 1,311 (7.3%), and 26 (0.15%) were epi-linked, laboratory-confirmed, and clinically compatible, respectively. Higher proportions of confirmed measles cases were observed among children aged >5 years and infants <9 months, compared with children aged 12–59 months (*p <* 0.001). A significantly high proportion of cases were enrolled from rural areas and were zero dose. Of 7,637 suspected measles cases tested, measles IgM antibodies were detected in 1,311 (17.2%) cases, with significantly more cases in 2023 than in 2024. Factors that were significantly associated with laboratory-confirmed measles cases in multivariate analysis were age groups 5 + years, under-vaccinated/unvaccinated, and unknown vaccination status. The estimated effectiveness of two doses in preventing laboratory-confirmed measles cases during the 2023 and 2024 outbreaks was 80.7%. Under-vaccinated and unvaccinated due to unavailability of vaccination services, stockouts, and missed opportunities were highlighted by stakeholders as contributing factors to measles outbreaks.

**Conclusion:**

The study revealed that the vast majority of confirmed cases occurred among inadequately immunized children, with limited access to vaccination services, driven by multiple underlying factors, emerging as a key contributor to measles outbreaks. These findings highlight the importance of implementing context-specific strategies to close immunity gaps.

## 1 Introduction

Measles virus (MeV) is a highly infectious virus that is transmitted primarily via aerosolized respiratory droplets (1) with a basic reproduction number (R0) of about 9–18, exceeding that of other viral diseases such as smallpox (R0 5–7) and polio (R0 4–13) (2). When routine immunization breaks down, measles is usually the first vaccine-preventable disease (VPD) to be detected; therefore, it is considered an indicator of the status of the stable vaccination programs (3).

The global burden of measles infection, including its associated morbidity and mortality, has declined substantially following the widespread introduction of measles-containing vaccines (MCVs). Since the 1960s, the live attenuated measles-containing vaccine (MCV) has demonstrated high effectiveness and confers durable, often lifelong immunity comparable to that acquired through natural infection (4). Based on the high R0, a population immunity of approximately 93–95%, is necessary to induce herd immunity and ensure measles transmission is interrupted (2, 5–7). The World Health Organization (WHO) recommends administration of the first dose of measles-containing vaccine (MCV) at 9 months of age in measles-endemic regions and at 12–15 months of age in non-endemic regions, followed by the second dose at 18 months of age (8). The second dose provides additional immunity to account for primary vaccination failure, which occurs in approximately 10–15% of those vaccinated with a first dose at 9 months of age (9). However, during measles outbreaks, MCV can be given as early as 6 months of age with a follow-up dose at 9 months of age (8). The goal of the current WHO-recommended vaccination schedule is to provide optimal vaccine efficacy following the decay of maternal antibodies, when the child is at increased risk of exposure to MeV (8).

According to the World Health Organization and the United Nations International Children’s Education Fund (WHO/UNICEF)—WUENIC—the global coverage of MCV1 was 84% in 2024, while that of MCV2 was 76%. In the African region, MCV1 coverage was 71% in 2024, while MCV2 coverage was 55%. Tanzania started the provision of monovalent MCV at 9 months of age in 1975, following WHO recommendations for the universal vaccinations globally (10). The National level coverage of MCV1 based on WUENIC has been reported to vary between 1980 and 2024. MCV1 coverage increased consistently from 46% in 1980 to 99% in 2013, then declined slightly to 97% in 2014. In 2021, the MCV1 coverage based on the WUENIC data was 80%, and the coverage stalled at 84% during 2022–2024 (11). Tanzania introduced MCV second dose (MCV2) in 2014, with coverage reported to range from 29% in 2014 to 74% in 2024 based on WUENIC data (11). During implementation of the two-dose MCV schedule, a reduction of measles cases of >90% from 53,459 cases in 2000–2013 to 1,324 in 2014–2021 (12) was documented.

Persistence of immunity gaps to measles due to unvaccination or under-vaccination has been documented, highlighting ongoing global measles outbreak challenges, including in Tanzania (13–15). A total of 874,099 measles cases were reported globally in 2019, 669,083 cases in 2023, and 476,412 cases in 2024; the African region contributed 70.8, 63.4, and 40% of the global cases reported in the respective years (12). The high proportion of cases in 2019 was due to the disruption of immunization activities by the coronavirus disease 2019 (COVID-19) pandemic, resulting in millions of children being unvaccinated or under-vaccinated against VPD (16, 17), with estimates of about 30 million children remaining under-protected against measles. In 2021, Tanzania accounted for 2% of the 24.7 million children reported globally who had not received the first dose of MCV and was ranked 10th among the countries with the highest number of zero-dose children (18, 19). Data from coverage surveys conducted post-Measles-Rubella Campaign in 2014, 2019, and 2024 reported coverage of 89, 88.25, and 81.5%, respectively, highlighting the accumulation of susceptible individuals (20, 21). Tanzania Demographic Health Survey (TDHS), which was conducted in 2022, documented that only 64% of children aged 24–35 months had received MCV2 (22). Immunity gaps resulting from under-vaccinated and unvaccinated children increased individuals’ susceptibility and led to large measles outbreaks caused by genotype B3 (23) in 2023 and 2024. Documenting the factors associated with laboratory-confirmed measles cases and exploring the causes of measles outbreaks through interviews and focus group discussions with key stakeholders is of paramount importance to ensure context-based strategies to prevent or minimize future measles outbreaks.

## 2 Methods

### 2.1 Study area, design, and duration

This was a cross-sectional study using the data from the National Measles Epidemiological Surveillance System, which includes 195 councils of the United Republic of Tanzania collected in 2023 and 2024. Surveillance of measles was conducted in accordance with the African Regional Guidelines for Measles and Rubella Surveillance (24). In addition, in-depth Key Informant Interviews (KIIs) with key stakeholders, program managers, partners, Regional Immunization and Vaccine Officers (RIVOs), District Immunization and Vaccine Officers (DIVOs), and healthcare workers were conducted, followed by a community focus group discussion (FGD) with caregivers to explore the factors associated with measles outbreaks. The qualitative data were collected across six regions, namely, Katavi (Mpanda and Mpimbwe District Councils [DCs]), Arusha (Arusha City and Karatu DCs), Tabora (Nzega and Kaliua DCs), Pwani (Kibaha and Mafia DCs), Mtwara (Nanyamba DC), and Pemba (Wete DC). The regions and districts were selected based on the number of measles cases during the 2023 and 2024 outbreaks.

### 2.2 Study population

Measles surveillance in Tanzania enrolls a person of any age presenting with fever and a generalized rash with onset within 30 days, regardless of vaccination status (vaccinated, unvaccinated, unknown vaccination status), at any health facility in the country. For the qualitative part, key stakeholders included program managers, partners (WHO, UNICEF), RIVOs and DIVOs, healthcare workers, and caregivers.

### 2.3 Sample size

No specific sample size was estimated. The study included all data in the National Measles Epidemiological Surveillance System database whose samples were collected and submitted to the National Public Health Laboratory (NPHL) for IgM testing. For the qualitative part, a total of 52 participants were identified for Key Informant Interviews (KIIs), and 10 community FGDs were conducted involving 5–10 participants per group (92 participants). The sample size for qualitative was enough to ensure broad coverage and achievement of the saturation, which usually occurs (9–17) for interviews or (4–8) for focus group discussions (25).

### 2.4 Sampling, inclusion, and exclusion criteria

A suspected measles case was defined as a person of any age with fever and generalized maculo-papular rash (24). Additionally, all cases with valid laboratory results and complete data on age, sex, residence, vaccination status, admission status, and clinical features were included in the subanalysis of factors associated with laboratory-confirmed measles cases. The analysis excluded cases that did not present with both fever and rash, those with rash onset occurring within 30 days of receiving a measles vaccine, and cases with missing key information, such as age, sex, or vaccination status.

For the qualitative part, purposive sampling was used to select study participants, who are directly involved in planning or implementing immunization services at the national and subnational levels, including caregivers of children. Purposive sampling was considered appropriate for this study because the selected participants shared specific characteristics relevant to the research objectives and were expected to provide rich, in-depth information on the topic.

### 2.5 Quantitative data collection

Once a case of measles or an outbreak was suspected at the facility, the notification was sent to the Council, Regional, and National surveillance teams. The notification aimed to ensure that surveillance protocols were adhered to and that the case investigation form (CIF) was accurately completed, followed by proper sample collection, labeling, storage, and transportation to the National level. CIF was used to collect basic data from all suspected Measles cases. Data collected included socio-demographic characteristics (age and sex), dates of rash onset, clinical symptoms, vaccination status, and council. Vaccination status data were collected from vaccination cards; when cards were unavailable, information was obtained from parental recall. The completed CIF with accurate information on the suspected case was submitted to the National Surveillance office at the EPI Mabibo, where the information was verified, and cases were assigned epidemiological (Epid) numbers. The same Epid number was used for case-based surveillance and laboratory surveillance.

### 2.6 Sample collection and laboratory procedures

Blood samples (5 mL per patient) were collected in a plain vacutainer tube (Becton Dickinson, Nairobi, Kenya) and centrifuged to obtain sera which were transported to the Immunization and Vaccine Development Program (VPD) surveillance unit for sorting and packaging, before being transported to the National Public Health Laboratory (ISO15189:2012 Accredited), Dar es Salaam, for testing (24). Sera were stored at −80 °C until processing. Prior to analysis, samples were allowed to reach room temperature (25 °C). Testing was performed according to standard operating procedures (SOPs) and the manufacturer’s instructions to detect measles-specific IgM antibodies using an indirect enzyme-linked immunosorbent assay (ELISA) (Euroimmun IgM ELISA, Seekamp 31, 23,560 Lübeck, Germany). The assay has a sensitivity of 100% and specificity of 98% in detecting measles-specific IgM antibodies (26).

### 2.7 Measles case definition and study variables

An outbreak was defined as the occurrence of three or more laboratory-confirmed measles cases among at least five suspected cases within a defined area and within 30 days of the onset. Measles cases were classified as laboratory-confirmed, epidemiologically linked, clinically compatible, or discarded, as previously described (24). The dependent variable in the analysis of factors was a laboratory-confirmed measles case, defined as positive IgM measles-specific antibodies. In contrast, the independent variables were age, sex, residence, vaccination status, admission status, and clinical symptoms.

### 2.8 Qualitative data collection

KIIs and FGDs with community members were conducted. Four individuals with experience conducting KIIs and FGDs were trained to conduct interviews and discussions to minimize investigator influence and reduce potential bias in the results. All interviews were conducted in Kiswahili, which is widely spoken and understood in Tanzania, to ensure participants’ comfort and comprehension.

KIIs and FGDs were guided by a semi-structured interview guide containing main questions and probes to help direct the conversation. Participants’ responses were audio-recorded using digital devices, and field notes were taken concurrently as a backup and to capture non-verbal expressions or contextual observations. In addition, socio-demographic information of the study participants was collected using a standardized form. Each KII took between 30 and 45 min, depending on the flow and depth of the discussion. Each FGD involved a homogenous group of participants and took between 1 and 2 h. All interview guides were pretested to ensure accuracy and relevance.

### 2.9 Data analysis

Data management and analysis were done using STATA 15. Data obtained from the case investigation forms were matched with the laboratory corresponding case, and a final case classification was determined in accordance with the WHO Measles Outbreak Guide (27). Categorical variables, such as sex, residence (urban and rural), vaccination status, and clinical symptoms, were summarized using proportions. In contrast, continuous variables, such as age and duration from onset to sample collection (days onset), were summarized as medians. Age was categorized into <9 months, 9–11 months, 1–4 years, 5–9 years, 10–14 years, 15–19 years, and ≥20 years to assess trends in laboratory-confirmed measles cases by age. To analyze the strength of association, age was further categorized into three cohorts: <5, 5–9.9, and ≥10 years. Measles incidence was calculated as the number of cases per 1,000,000 population and stratified by age group (<1, 1–4, 5–9, 10–14, 15–19, and 20 + years), sex, and geographical area (regions, administrative level 1). Logistic regression analysis was conducted among cases aged ≥9 months with documented vaccination status (two doses, single dose, and zero dose) to identify factors associated with zero-dose status (i.e., receipt of no measles-containing vaccine [MCV]). Additionally, logistic regression analysis was performed to estimate the strength of association between independent variables and laboratory-confirmed measles cases. Collinearity between age and vaccination status was assessed and found not to influence the final model significantly.

Variables that were statistically significant in the univariate analysis were included in the multivariable logistic regression model, with adjustment for age and sex. Adjusted odds ratios (aORs) and their 95% confidence intervals (CIs) were reported, and a *p*-value of <0.05 was considered statistically significant.

The estimated vaccine effectiveness (VE) of a single MCV dose and two MCV doses in protecting against laboratory-confirmed measles cases during outbreaks was calculated by comparing the proportion of laboratory-confirmed measles cases among vaccinated individuals with one or two doses with that among unvaccinated individuals who submitted samples. Attack rate among vaccinated (ARV) and attack rate among unvaccinated (ARU) were used to calculate the vaccine effectiveness estimates (VE) = (ARU – ARV)/ ARU × 100% as previously described (28).

For qualitative data, thematic analysis was used to analyze the data, following Braun and Clarke’s six-step guidelines (29). This method was selected for its strength in systematically identifying, analyzing, and reporting patterns and recurring themes in qualitative data. The analysis process included the following steps: transcription and translation, familiarization with the data, generating initial codes, searching for themes, reviewing themes, defining and naming themes, and interpreting the data.

## 3 Results

### 3.1 Overall description of enrolled cases and measles classifications in relation to age, residence, and vaccination status

**Figure 1:**
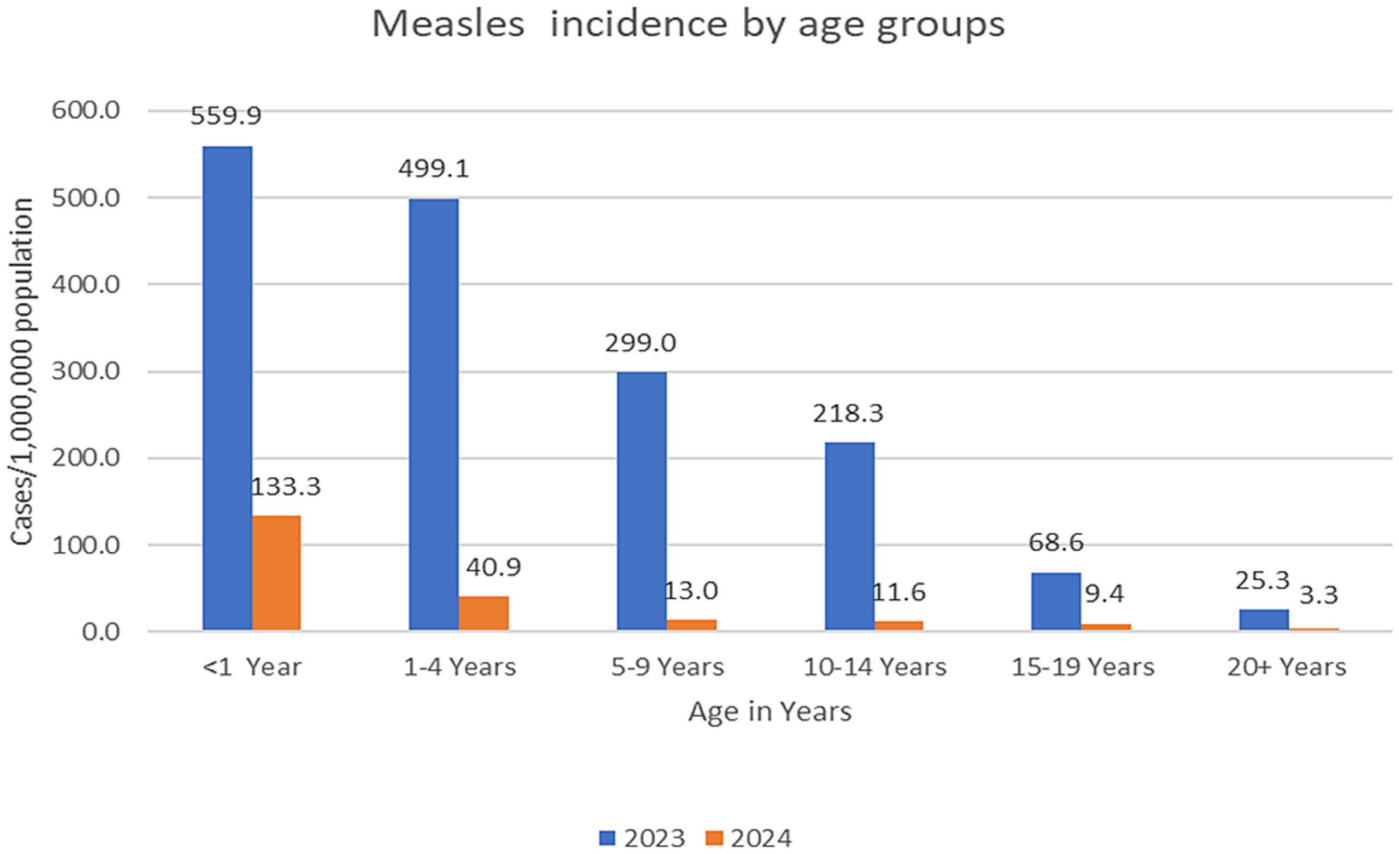
Incidence of confirmed measles cases per 1,000,000 population by age groups and years.

A total of 17,902 suspected cases were enrolled in 2023 and 2024; of these, 13,459 (75.2%) were enrolled in 2023 and 4,443 (24.8%) in 2024. The median age of enrolled suspected cases was 48 months (interquartile range [IQR]: 21–108). Cases enrolled in 2024 had a significantly lower median age (36 months, IQR: 18–60) compared to cases enrolled in 2023 (49 months, IQR: 24–112) (*p <* 0.001). Of the 17,902 suspected cases, 11,576 (64.7%) were confirmed measles cases, of which 1,311 (7.3%) were laboratory-confirmed cases, 10,239 (57.2%) were epidemiologically linked cases, and 26 (0.15%) were classified as clinically compatible cases. The overall incidence rate declined markedly from 163.9 cases per 1,000,000 population in 2023 to 15.4 cases per 1,000,000 population in 2024 (*p <* 0.001). Age-specific analysis showed that the highest incidence occurred among children aged <1 year, with 559.9 cases per 1,000,000 population in 2023 and 133.3 cases per 1,000,000 population in 2024. This was followed by children aged 1–4 years, who consistently exhibited the second-highest incidence in both years (Figure 1). By sex, males had a higher incidence rate than females (177.4 vs. 164.5 cases per 1,000,000 population in 2023 and 16.9 vs. 15.7 per 1,000,000 population in 2024).

**Figure 2:**
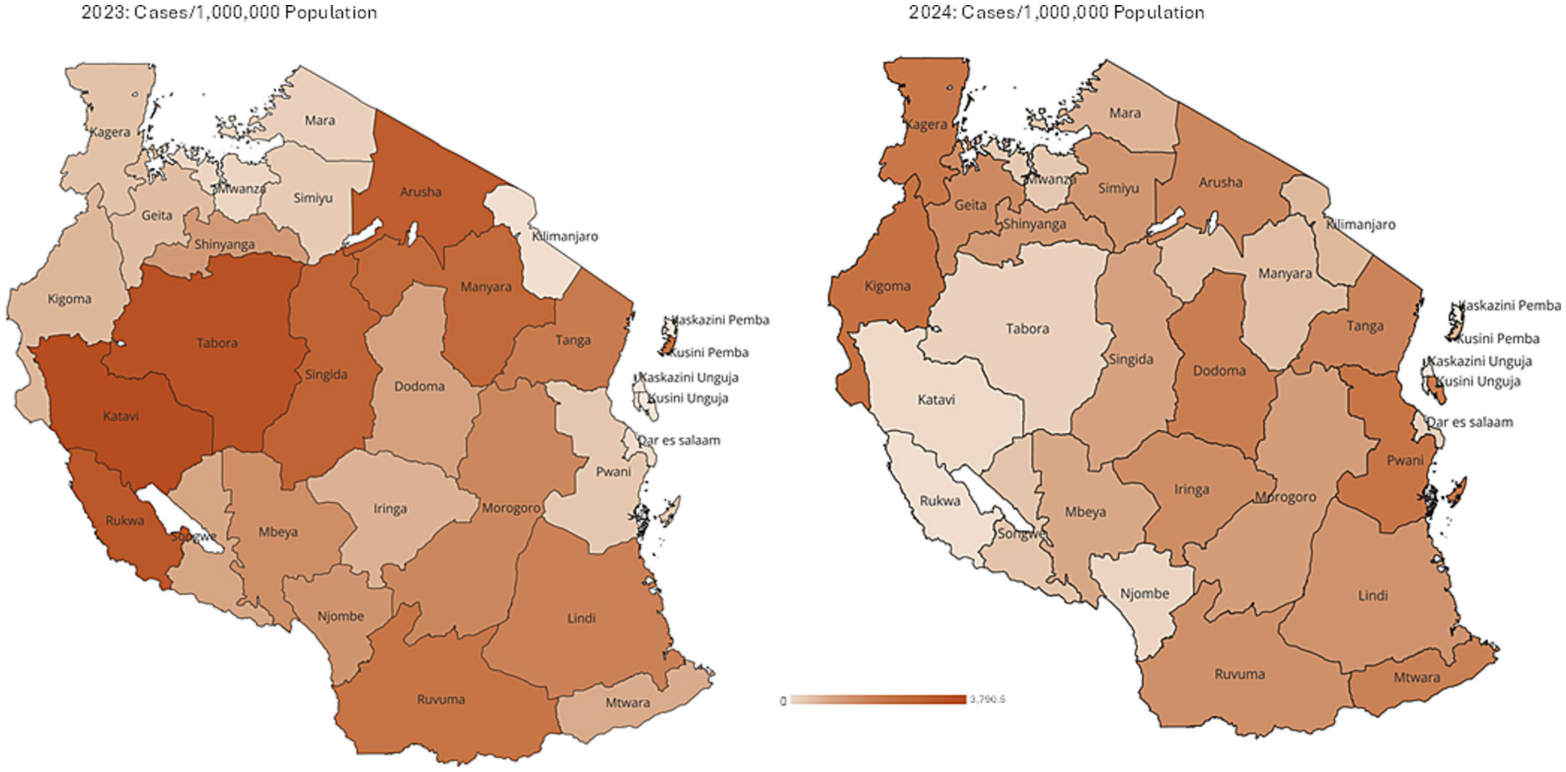
Maps showing incidence rate by regions, ranging from 0 to 3,790 cases per 1,000,000 population.

Geographically, substantial regional variation was observed. In 2023, Katavi recorded the highest incidence rate (3,790 cases per 1,000,000 population), followed by Tabora (1,218.76 cases per 1,000,000 population). Overall, 7 (22.6%) regions reported incidence rates exceeding 100 cases per 1,000,000 population in 2023. In 2024, the majority of the regions experienced a notable decline in incidence compared to 2023. However, Kigoma and Pwani showed an increase in incidence rates, rising to 167.45 and 86.54 cases per 1,000,000 population, respectively, during 2024 (Figure 2).

**Figure 3:**
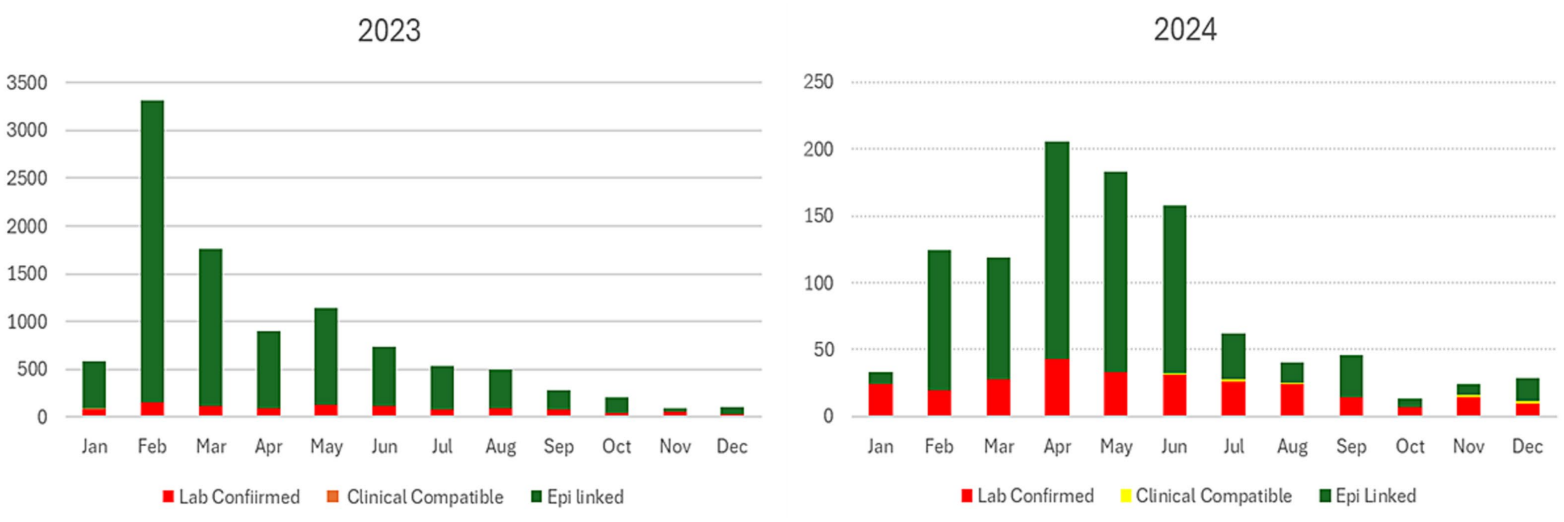
Incidence and trend of measles cases by month for 2023 and 2024.

Overall, 7,637 samples (2023 = 3,957; 2024 = 3,680) were tested to confirm laboratory-confirmed measles cases. The peak of measles cases was noted in February 2022 and April 2024 (Figure 3).

**Figure 4:**
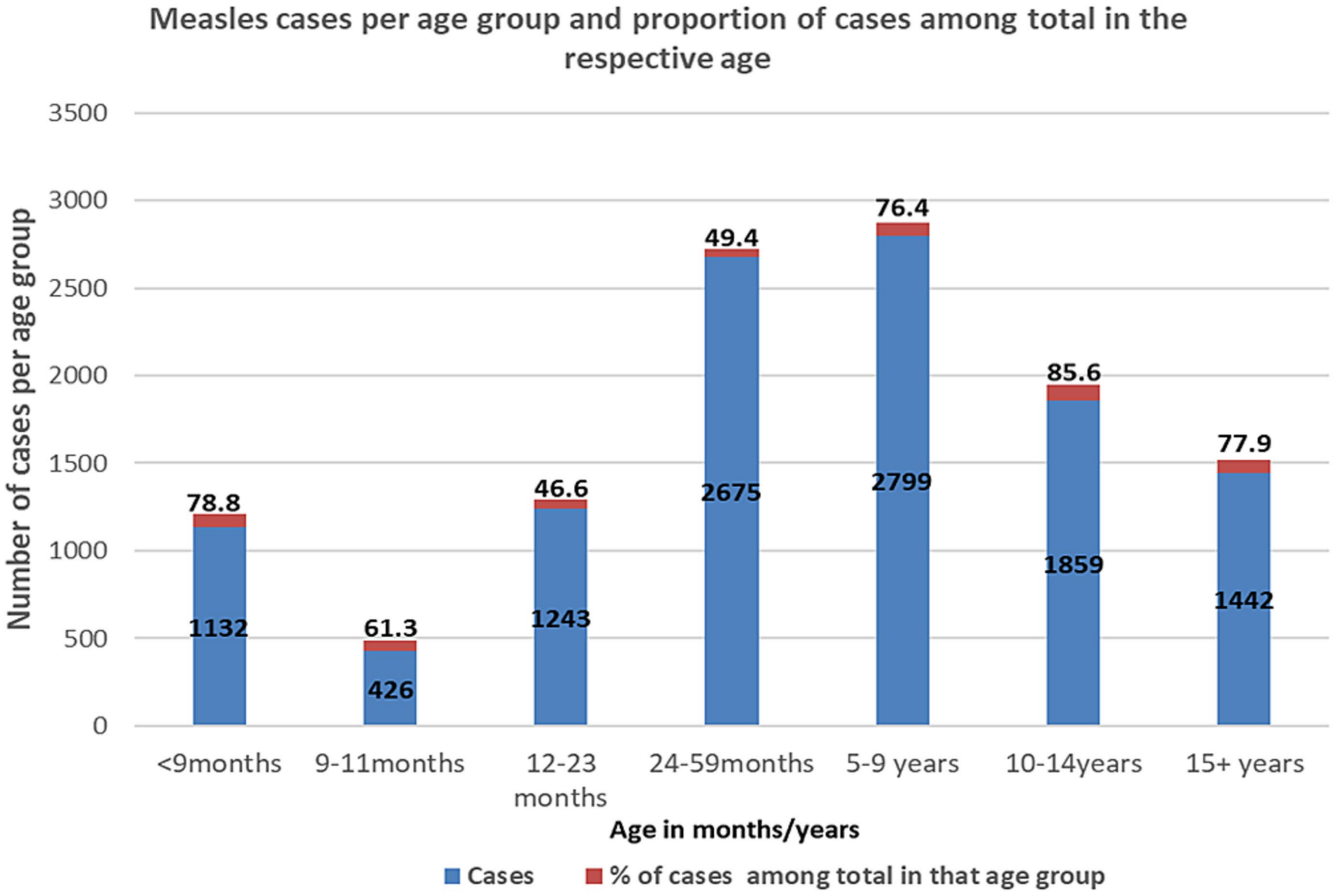
Age in relation to number of confirmed measles cases and proportions of cases.

Regarding age, the proportions of confirmed measles cases were 78.8, 61.3, 46.6, 49.4, 76.4, 85.6, and 77.9% for age groups <9 months, 9–11 months, 12–23 months, 24–59 months, 5–9 years, 10–14 years, and 15 + years, respectively. Significantly higher proportions of cases were observed in age groups above 5 years and below 9 months, compared with the age 12–59 months group (Figure 4).

Regarding the vaccination status of the suspected cases, in 2023, 32.7% were zero dose, 15.9% had received one dose, 17.7% had received two doses, 9.4% were not eligible, and 24.2% had an unknown vaccination status. In 2024, 7.7% were zero dose, 18.3% had received one dose, 50.7% had received two doses, 5.7% were not eligible, and 17.7% had unknown vaccination status. A significantly higher proportion of zero-dose cases was observed in 2023 (*p <* 0.001). Among 15,510 suspected cases from rural areas, 10,659 (68.7%) were confirmed measles cases, compared to 917 (38.3%) out of 2,392 cases from urban areas (*p <* 0.001), and this difference remained significant when stratified by year of outbreak. Additionally, a significantly higher proportion of cases from rural areas were zero dose compared to those from urban areas (28.2%, *n =* 4,376 vs. 15.6%, *n =* 373; *p <* 0.001).

### 3.2 Socio-demographic and clinical characteristics of the suspected cases submitted samples for laboratory confirmation

A total of 7,637 samples were collected for laboratory confirmation of measles cases. The median age was 36 months (IQR: 20–71). There was an almost equal distribution by sex. Regarding vaccination status, 51.2% of the investigated cases had received two doses of MCV, 22.7% had received one dose, 14.9% had unknown vaccination status, and 5.3% had not received any dose of MCV. A high proportion of cases was managed as outpatients (92.6%). In addition to fever and rashes, 86.7, 74.2, 32.6, 5.2, and 4.8% of cases had cough, running nose, red eyes, joint pains, and swollen lymph nodes, respectively (Table 1).

**Table 1:** Socio-demographic and clinical characteristics of 7,637 participants.

Variable	*N*	%
Median (IQR) age in months	7,637	36: IQR (20–71)
Age category (years)		
<5	5,370	70.3
5–9.99	1,188	15.6
>10	1,079	14.1
Sex		
Female	3,768	49.3
Male	3,869	50.7
Year sample collected		
2023	3,957	51.8
2024	3,680	48.2
Residence		
Urban	1,749	22.9
Rural	5,888	77.1
Vaccination		
Two doses	3,909	51.2
One dose	1,733	22.7
Zero dose	405	5.3
Unknown	1,140	14.9
Not eligible	450	5.9
Median (IQR) days-onset	7,637	3: IQR (2–4)
Admission status		
Outpatient	7,070	92.6
Inpatient	567	7.4
Cough		
No	1,019	13.3
Yes	6,618	86.7
Running nose		
No	1,967	25.8
Yes	5,670	74.2
Joint pain		
No	7,239	94.8
Yes	398	5.2
Red eyes		
No	5,145	67.4
Yes	2,492	32.6
Swollen lymph nodes		
No	7,270	95.2
Yes	367	4.8

**Figure 5:**
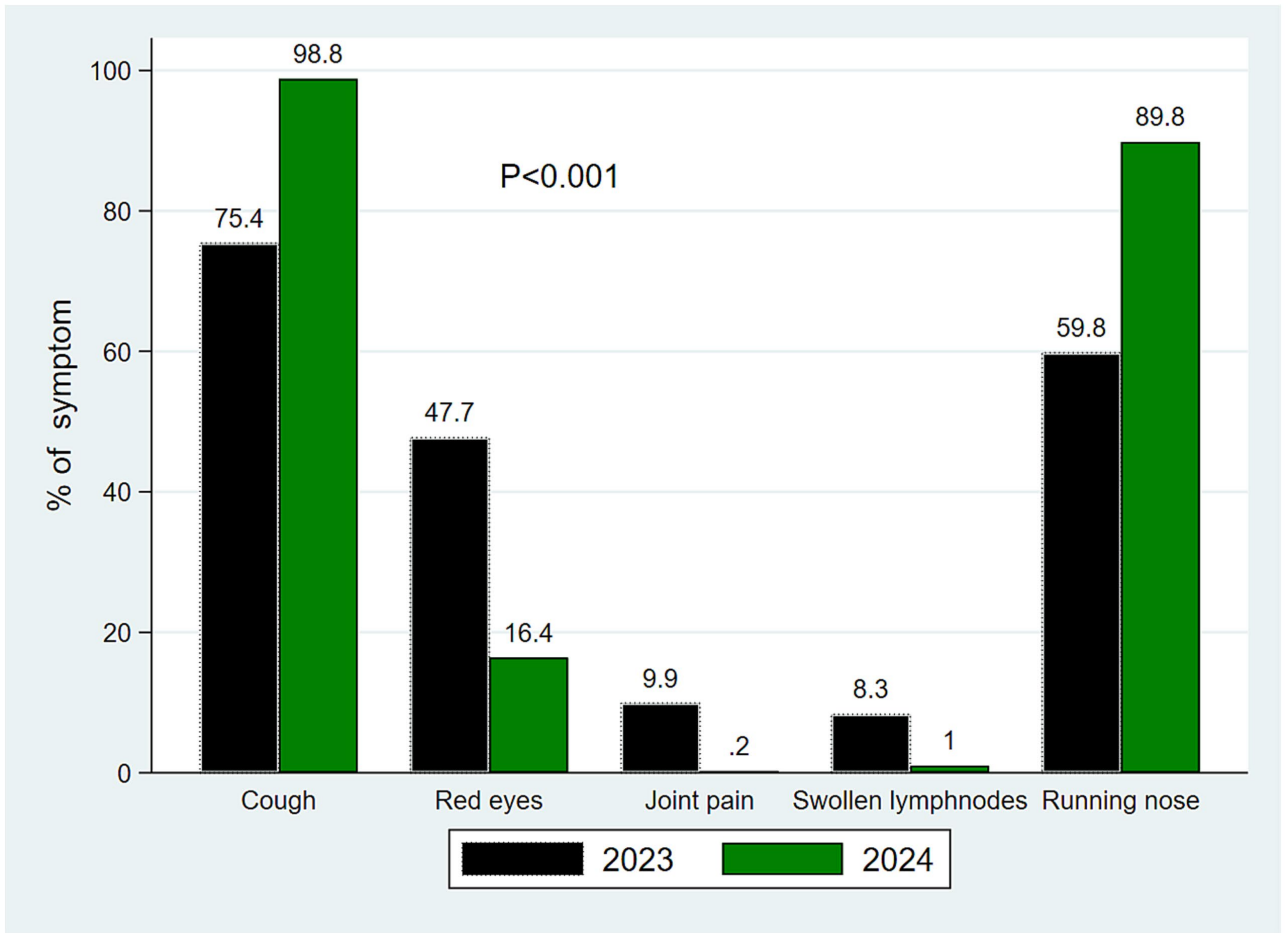
Distribution of symptoms by year of sample collection.

Joint pain, red eyes, and swollen lymph nodes were significantly more common in 2023 cases than in 2024 cases, while cough and a running nose were more common in 2024 cases (Figure 5). The median duration in days from symptom onset to sample collection was 3 (IQR: 2–4).

### 3.3 Vaccination status and admission status among laboratory-investigated cases

Admission status varied significantly by vaccination category. A total of 134 (3.4%) of 3,909 children vaccinated with two doses, 110 (6.4%) of 1,733 vaccinated with one dose, 109 (26.9%) of 405 unvaccinated children, 164 (14.4%) of 1,140 with unknown vaccination status, and 50 (11.1%) of 450 children not eligible for MCV were admitted (*p <* 0.001). The odds of admission were 10.4 times higher among unvaccinated children compared to those who had received two doses of MCV.

### 3.4 Factors associated with MCV zero dose among laboratory-confirmed cases aged ≥ 9 months

A total of 6,040 laboratory-confirmed cases aged ≥9 months were subanalyzed to identify factors associated with MCV zero-dose status. Higher proportions of zero-dose cases were observed among those aged ≥5 years than those aged <5 years (*p <* 0.001). In addition, 7.2% of cases from rural areas were zero dose, compared with 4.9% of cases from urban areas (*p* = 0.004). In the multivariate analysis, factors independently associated with zero-dose status included age ≥5 years (*p <* 0.001), rural residence (p = 0.004), inpatient status (*p <* 0.001), presence of red eyes (*p <* 0.001), and occurrence during 2023 (*p <* 0.001) (Table 2).

**Table 2:** Factors associated with zero dose (MCV unvaccinated) among 6,040 cases aged ≥ 9 months with vaccination status.

Variable	*N*	Univariate			Multivariate	
		(MCV) Zero dose positive: *n* (%)	OR (95% CI)	*p*-value	aOR (95% CI)	*p*-value
Age category (years)						
<5	4,750	227 (4.8)	1			
5–9.99	894	97 (10.8)	2.42 (1.88–3.11)	<0.001	1.89 (1.45–2.46)	<0.001
>10	396	81 (20.5)	5.12 (3.87–6.76)	<0.001	3.68 (2.72–4.97)	<0.001
Sex						
Female	2,977	197 (6.6)	1		1	
Male	3,063	208 (6.8)	1.02 (0.84–1.25)	0.788	1.07 (0.87–1.33)	0.493
Residence						
Urban	1,334	66 (4.9)	1			
Rural	4,706	339 (7.2)	1.49 (1.13–1.95)	0.004	1.51 (1.14–2.01)	0.004
Year						
2024	3,025	90 (2.9)	1			
2023	3,015	315 (10.4)	3.80 (2.99–4.83)	<0.001	2.15 (1.65–2.81)	<0.001
Admission status						
Outpatient	5,687	296 (5.2)	1			
Inpatient	353	109 (30.9)	8.13 (6.30–10.49)	<0.001	3.83 (2.88–5.09)	<0.001
Cough						
No	800	41 (5.1)	1			
Yes	5,240	364 (6.9)	1.38 (0.99–1.92)	0.056		
Running nose						
No	1,519	97 (6.4)	1			
Yes	4,521	308 (6.8)	1.07 (0.84–1.35)	0.565		
Joint pain						
No	5,802	362 (6.2)	1			
Yes	238	43 (18.1)	3.31 (2.34–4.68)	<0.001	1.39 (0.93–2.07)	0.104
Red eyes						
No	4,153	180 (4.33)	1			
Yes	1,887	225 (11.9)	2.98 (2.43–3.66)	<0.001	1.66 (1.32–2.09)	<0.001
Swollen lymph nodes						
No	5,772	369 (6.4)	1			
Yes	268	36 (13.4)	2.27 (1.57–3.27)	<0.001	1.18 (0.784–1.79)	0.418

### 3.5 Laboratory results (measles IgM and rubella IgM)

**Figure 6:**
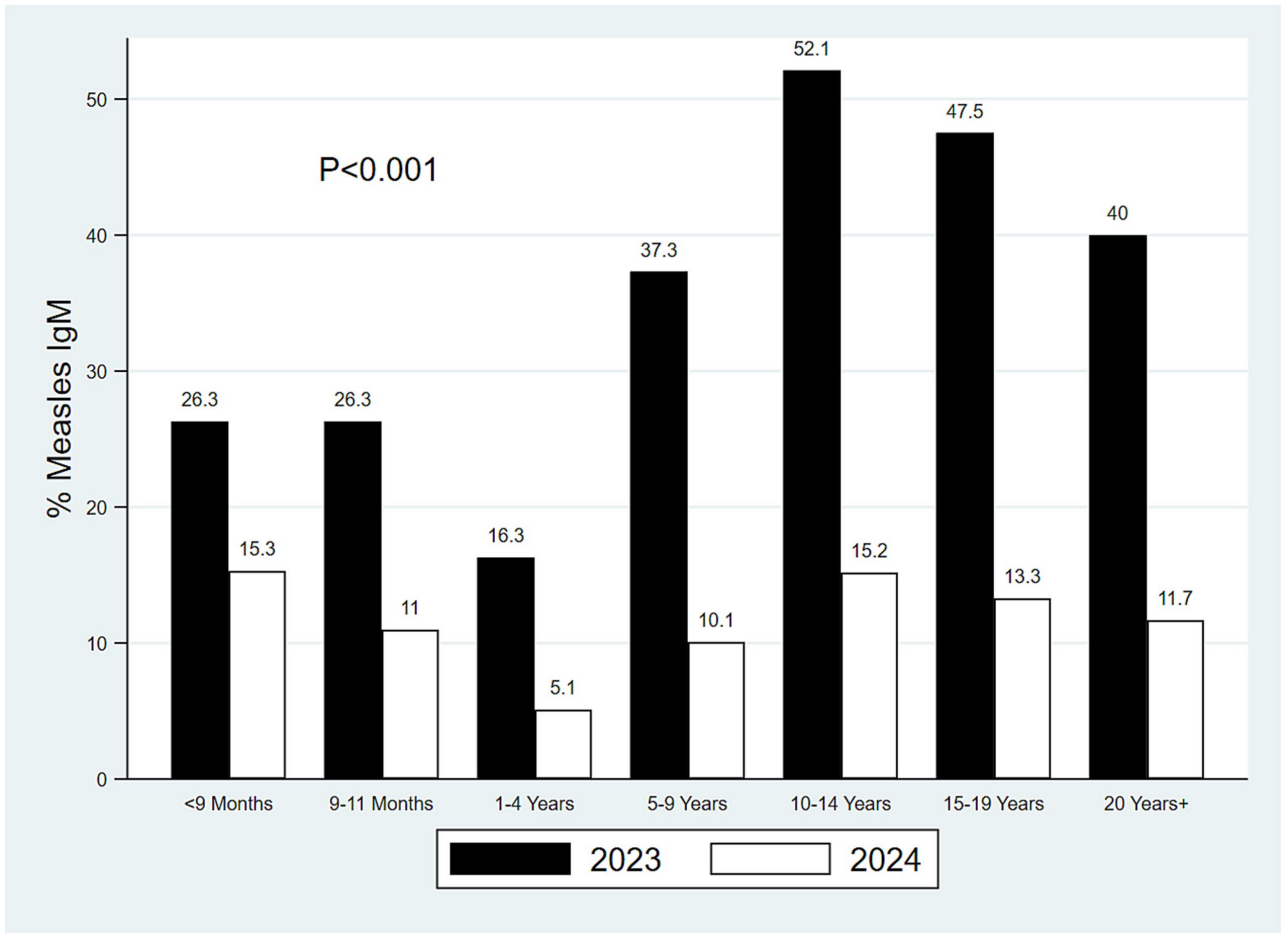
Laboratory-confirmed measles cases by age for 2023 and 2024.

Measles-specific IgM antibodies were detected in 1311 (17.2, 95% CI: 16.1–17.8) of 7,637 non-repetitive serum samples tested. The confirmed laboratory measles cases were significantly higher in 2023 than in 2024. Of the 3,957 cases enrolled in 2023, 1,037 (26.2%) were confirmed measles cases compared with 274 (7.4%) of the 3,680 cases enrolled in 2024 (*p <* 0.001). Furthermore, it was observed that 2,202 (59.8%) of the cases enrolled in 2024 received two doses of MCV compared to 1,707 (43.1%) of the cases enrolled in 2023 (*p <* 0.001). In addition, when compared by age category, the proportions of the laboratory-confirmed measles cases were significantly lower in 2024 than in 2023 (Figure 6).

### 3.6 Factors associated with laboratory-confirmed measles cases among suspected cases

Factors that were significantly associated with laboratory-confirmed measles cases on univariate analysis were age group 5–9.9 years (OR: 2.78, 95% CI: 2.39–3.24), ≥ 10 years (OR: 3.76, 95% CI: 3.23–4.38), rural residence (OR: 1.18, 95% CI: 1.02–1.37), those vaccinated with only one dose (OR: 1.65, 95%:1.38–1.95), unvaccinated (OR: 8.90, 95% CI: 7.11–11.13), unknown vaccination status (OR: 6.06, 95% CI: 5.14–7.13), not eligible for vaccination, that is, <9 months of age (OR:2.99, 95% CI: 2.33–3.82), and those managed as inpatients (OR: 8.86, 95% CI: 7.40–10.61). Regarding symptoms, cough (*p* = 0.033), joint pain (*p <* 0.001), red eyes (*p <* 0.001), and swollen lymph nodes (*p <* 0.001) were also associated with laboratory-confirmed measles cases on univariate analysis.

On multivariate analysis, age group 5–9.9 years (OR: 2.06, 95% CI: 1.71–2.47), ≥ 10 years (OR: 1.85, 95% CI: 1.49–2.28), those vaccinated with only one dose (OR: 1.56, 95%: 1.30–1.88), unvaccinated (OR: 4.97, 95% CI: 3.87–6.38), unknown vaccination status (OR: 3.27, 95% CI: 2.65–4.03), not eligible for vaccination, that is, <9 months of age (OR: 3.24, 95% CI: 2.49–4.23), those managed as inpatients (OR: 4.22, 95% CI: 3.44–5.17), joint pain (OR: 1.96, 95% CI: 1.50–2.55), and red eyes (OR: 2.28, 95% CI: 1.98–2.62) remained significantly associated with laboratory-confirmed measles (Table 3).

**Table 3:** Factors associated with laboratory-confirmed measles cases (*N =* 7,637).

Variable	*N*	Univariate			Multivariate	
		Measles IgM positive: *n* (%)	OR (95% CI)	*p*-value	aOR (95% CI)	*p*-value
Age category (years)						
<5	5,370	630 (11.7)	1			
5–9.99	1,188	321 (27.0)	2.78 (2.39–3.24)	<0.001	2.06 (1.71–2.47)	<0.001
>10	1,079	360 (33.4)	3.76 (3.23–4.38)	<0.001	1.85 (1.48–2.29)	<0.001
Sex						
Female	3,768	669 (17.7)	1		1	
Male	3,869	642 (16.6)	0.92 (0.81–1.03)	0.182	0.93 (0.81–1.06)	0.297
Residence						
Urban	1,749	268 (15.3)	1			
Rural	5,888	1,043 (17.7)	1.18 (1.02–1.37)	0.02	1.14 (0.97–1.34)	0.102
Vaccination						
Two doses	3,909	353 (9.0)	1			
One dose	1,733	243 (14.0)	1.65 (1.38–1.95)	<0.001	1.56 (1.30–1.88)	<0.001
Zero dose	405	190 (46.9)	8.90 (7.11–11.13)	<0.001	4.97 (3.87–6.38)	<0.001
Uknown	1,140	421 (37.6)	6.06 (5.14–7.13)	<0.001	3.27 (2.65–4.03)	<0.001
Not eligible	450	103 (22.9)	2.99 (2.33–3.82)	<0.001	3.24 (2.49–4.23)	<0.001
Days-onset	1,311	3: IQR (2–5)	0.99 (0.997–1.001)	0.737		
Admission status						
Outpatient	7,070	978 (13.8)	1			
Inpatient	567	333 (58.7)	8.86 (7.40–10.61)	<0.001	4.22 (3.44–5.17)	<0.001
Cough						
No	1,019	151 (14.8)	1			
Yes	6,618	1,160 (17.5)	1.22 (1.01–1.46)	0.033	1.17 (0.95–1.42)	0.121
Running nose						
No	1,967	340 (17.2)	1			
Yes	5,670	971 (17.1)	0.98 (0.86–1.13)	0.871		
Joint pain						
No	7,239	1,136 (15.7)	1			
Yes	398	175 (44.0)	4.21 (3.42–5.18)	<0.001	1.96 (1.51–2.55)	<0.001
Red eyes						
No	5,145	570 (11.1)	1			
Yes	2,492	741 (29.7)	3.39 (3.00–3.83)	<0.001	2.28 (1.96–2.62)	<0.001
Swollen lymph nodes						
No	7,270	1,203 (16.5)	1			
Yes	367	108 (29.4)	2.10 (1.66–2.65)	<0.001	0.85 (0.63–1.15)	0.414

### 3.7 Vaccine effectiveness

The estimated effectiveness of two-dose and a single-dose MCV to prevent laboratory-confirmed measles cases in 2023 and 2024 outbreaks using the screening method was 80.7 and 70.1%, respectively, with the assumption that unvaccinated individuals were only those with documented vaccination status. The effectiveness decreased with increasing age: 81.6% (1–4 years), 79.1% (5–9 years), and 63.7% (10–14 years), and 75.2, 45.2, and 45.7% for MCV2 and MCV1, respectively.

### 3.8 Qualitative results

A total of 52 Key Informant Interviews (Table 4) and 10 FGD were conducted. The FGD included 92 participants, with 54.2% aged 18–30 years and 45.8% aged 31 years or older. Two main themes were identified: (i) causes of the measles outbreaks and (ii) reasons for unvaccination/under-vaccination (Table 5).

**Table 4:** Key informant interviews by category.

Category	*N*
Program managers/officers/vaccine focal persons	7
Grant coordinator	1
Data manager	1
Regional immunization officers (RIVOs)	7
District immunization officers (DIVOs)	16
Partners	4
Healthcare workers	16
Total	52

**Table 5:** Causes of measles outbreak and reasons for unvaccination/under-vaccination.

Themes	Sub-theme	Sub-sub-theme
Causes of the measles outbreak	Unvaccinati on/under-vaccination	
	Reduced vaccine potency/cold chain failures	
Reasons for unvaccination/under-vaccination	Health system factors	Vaccine stockouts
		Missed opportunities during health visits Accessibility and geographical barriers Older age children are missed by routine services
	Caregiver factors	Lack of awareness and caregiver knowledge
		Competing caregiving responsibilities Misconceptions and fear of side effects

### 3.9 Theme 1: causes of measles outbreak

Participants across all study sites reported that the causes of the measles outbreaks were largely linked to the presence of unvaccinated and under-vaccinated children, as well as reduced vaccine potency due to cold chain failures. These factors were found to be influenced by challenges of both caregivers and the health system.

#### 3.9.1 Unvaccinated and under-vaccinated children

Across all regions, respondents consistently reported that many children had either missed their measles doses or were never vaccinated, leaving large pockets of susceptible children. Managers attributed this to poor follow-up and weak screening mechanisms during routine and catch-up activities.

A district immunization officer noted that:

*“In our district, many children miss the measles vaccine because their parents do not bring them on time. When one child gets measles, it spreads quickly among unvaccinated children” (KII003M).*

Health workers echoed this challenge, explaining that some children receive initial vaccines but miss the measles dose due to weak follow-up systems:

*“Some children receive initial vaccines but miss the measles dose. Older children who were not screened properly are left unvaccinated” (KII012, Healthcare worker).*

Caregivers confirmed these gaps, sharing experiences of missing vaccination appointments:

*“My child received all vaccines except measles. I didn’t know the exact date, so I missed it” (FGD2, Male caregiver).*

#### 3.9.2 Reduced vaccine potency/cold chain failures

Several participants described weaknesses in cold chain management, including unreliable refrigeration, poor temperature monitoring, and prolonged exposure of vaccines to ambient temperatures. These challenges were particularly pronounced in remote areas, which could potentially reduce vaccine effectiveness.

A health worker explained:

*“Sometimes the fridge fails or the vaccine is left out too long. We worry that some doses may lose potency, so children do not get proper protection” (KII008, Health Worker).*

A regional immunization officer similarly observed that:

*“In a few facilities, the cold chain is weak. Vaccines for measles may not be potent by the time they reach the child, especially in remote areas” (KII005M).*

### 3.10 Theme 2: reasons for unvaccinated/under-vaccinated children for measles

Participants identified multiple reasons contributing to the high number of unvaccinated and under-vaccinated children across the study areas. These included vaccine stockouts, missed opportunities during health visits, accessibility and geographical barriers, lack of awareness and caregiver knowledge, misconceptions and fear of side effects, older age children missed by routine services, and competing caregiving responsibilities.

#### 3.10.1 Vaccine stockouts

Frequent and prolonged stockouts of measles vaccines were a major barrier to full immunization coverage. Respondents explained that inconsistent supplies disrupted scheduled outreach and facility-based immunization sessions, leaving many children unvaccinated or delaying their doses.

A regional immunization officer emphasized:

*“Stock-outs and weak cold chains contribute to children being under-vaccinated for measles” (KII005M).*

A caregiver from Karatu shared a direct experience:

*“The nurse told me the vaccine was not given because it had been stored too long and might not work” (FGD 01, Female caregiver, Karatu).*

#### 3.10.2 Missed opportunities during health visits

Both health workers and caregivers reported that children visiting facilities for other services, such as growth monitoring or vitamin A supplementation, were not consistently screened for missed measles doses. This created repeated missed opportunities to vaccinate eligible children.

In some cases, caregivers were turned away by health workers due to strict vaccination days or rigid procedures:

*“I took my child for a check-up, but they said the measles vaccine is only given on certain days, so my child missed it” (FGD3, Female caregiver, Kaliua).*

*“I brought my child on a different day, but the nurse said they only give vaccines on Tuesdays. I had to go back home” (FGD01, Female caregiver, Karatu).*

#### 3.10.3 Accessibility and geographical barriers

Respondents from rural districts reported that long distances, poor road conditions, and seasonal rains made access to vaccination services difficult, especially for communities dependent on outreach.

*“In remote villages, families cannot reach the facility on vaccination days, and children remain unvaccinated” (KII007M).*

*“Some villages are located far from the health facility, and the roads are bad. During the rainy season, the car cannot reach those areas, and the outreach is postponed” (KII010, Healthcare Worker).*

*“When it rains heavily, the rivers overflow, and mothers can’t cross to come for vaccination. They end up missing the measles dose” (KII011, Healthcare Worker).*

#### 3.10.4 Lack of awareness and low caregiver knowledge

Caregivers’ limited knowledge of vaccination schedules and the importance of the measles vaccine also contributed to under-immunization. Some believed that vaccination was unnecessary once a child appeared healthy or had received early-life doses.

*“Some parents do not know the importance of measles vaccination, or they forget the schedule, so children miss doses” (KII014, Healthcare worker).*

*“I thought my child was healthy and didn’t need the vaccine urgently” (FGD03_Female caregiver, P5).*

#### 3.10.5 Misconceptions and fear of side effects

A few participants pointed out that rumors and misconceptions discouraged timely vaccination. Fear of side effects and misinformation circulating within communities led to delayed or missed doses.

*“Some parents fear side effects or believe the vaccine can harm their child” (KII009, Health Worker).*

*“Some neighbours said the measles vaccine can make children sick, so I waited before taking my child” (FGD03_ Female caregiver, P6).*

#### 3.10.6 Older age children missed by routine services

Health workers observed that microplans and routine immunization schedules often prioritized children under 1 year, leaving older children unvaccinated, especially against measles.

*“Our microplans often focus on under-one child, so children above 1 year may be missed for measles” (KII006M).*

*“Older children coming for vaccination are not always screened systematically, leading to missed measles doses” (KII013, Health Worker).*

#### 3.10.7 Child spacing and competing care responsibilities

Across several districts, both health workers and caregivers highlighted that closely spaced pregnancies and the demands of caring for multiple young children often contributed to missed or delayed measles vaccination. Mothers who had recently delivered another baby were frequently overwhelmed with caregiving duties, resulting in older children, especially those around 1 year of age, missing their scheduled measles second dose.

Key Informant Interviews with district immunization officers and health workers revealed that child spacing issues commonly led to missed vaccination appointments. Health workers observed that when mothers had another baby soon after the first, their attention and mobility were limited. They often postponed taking the older child for vaccination due to the demands of caring for an infant, household chores, or fatigue after delivery.

*“Some mothers get another baby before the older one finishes all vaccines. When they have a newborn, they find it difficult to go out, so the older child misses the measles dose” (KII011, Healthcare Worker).*

*“We have seen many cases where mothers with small babies forget or delay taking the older one for vaccination. It is not that they refuse it’s the challenge of balancing both children” (KII004m).*

*“Child spacing affects compliance. A mother who delivered recently might not manage to bring the older child to the clinic because she is still recovering or nursing” (KII006m).*

## 4 Discussion

Large measles outbreaks occurred in the United Republic of Tanzania in 2023 and 2024, with peaks in reported cases observed in February of both years. Key stakeholders identified several contributing factors to these outbreaks, including a high proportion of unvaccinated and under-vaccinated children, missed opportunities, and potential reductions in vaccine potency due to cold chain failures. In addition, health system constraints limiting the availability and accessibility of vaccination services, together with caregiver-related factors such as inadequate child spacing, misconceptions about vaccination, lack of awareness, and low caregiver knowledge, were highlighted as critical drivers of under-vaccination and non-vaccination as reported by a study from India (30). Notably, a significantly higher proportion of laboratory-confirmed measles cases and zero-dose children were reported from rural areas, underscoring persistent geographical disparities in access to immunization services as previously reported (31).

Under-vaccination may also have been exacerbated by the COVID-19 pandemic, during which declines in coverage for multiple antigens were observed, alongside an increase in the number of zero-dose children. In Tanzania, the number of districts with diphtheria tetanus toxoid and pertussis (DTP3) coverage below 80% increased from 8 in 2019 to 20 in 2021, indicating substantial disruption of routine immunization services (32). Furthermore, WHO/UNICEF Estimates of National Immunization Coverage (WUENIC) ranked Tanzania among the top-20 countries with the highest numbers of unvaccinated and under-vaccinated children, with nearly half a million affected in 2021.

Being unvaccinated has consistently been associated with an increased risk of laboratory-confirmed measles across multiple studies, underscoring the critical importance of sustaining high coverage of measles-containing vaccines (MCV) for effective control and eventual elimination of the disease (31, 33, 34).

The overall incidence rate of measles was significantly higher in 2023 (163.9 per 1,000,000 population) than in 2024 (15.4 per 1,000,000 population), with a correspondingly higher number of laboratory-confirmed cases reported in 2023. The elevated burden observed in 2023 may be partly explained by lower vaccination coverage, whereas in 2024, a greater proportion of cases had received two doses of measles-containing vaccine (MCV). Additionally, the nationwide Supplemental Immunization Activities (SIAs) conducted in 2024, immunity acquired following exposure during the 2023 measles outbreak, and catch-up vaccination campaigns implemented in response to ongoing outbreaks likely contributed to the substantial decline in measles transmission in 2024, reducing laboratory-confirmed cases by more than 70% and the overall incidence rate by approximately 90.6%. Consistent with findings from previous studies in Yemen, higher incidence rates were observed among males and children under 5 years of age (35).

The proportion of laboratory-confirmed measles cases observed in this study falls within the range reported in previous studies, which have documented proportions ranging from 4% in Ethiopia to 44% in the United Kingdom among samples submitted for testing (36–38). These variations are likely attributable to differences in the sensitivity and specificity of the clinical criteria used to define suspected measles cases (39, 40).

In this analysis, the odds of laboratory-confirmed measles were significantly higher among children under 1 year, those aged 5–9.9 years, and those aged 10 years or older compared to children aged 1–4 years. This finding may be explained by lower proportions of individuals who had received two doses of MCV in the older age groups relative to the 1–4-year age group, as reported in a previous study (41). The observation of a high incidence of laboratory-confirmed cases among infants below 1 year of age highlights a critical immunity gap, as these infants are typically not yet eligible for routine measles vaccination. In such cases, protection relies on herd immunity; therefore, low population-level vaccination coverage increases the risk of exposure and infection in this vulnerable group.

All suspected cases presented with fever and rash; however, significantly more cases in 2023 exhibited additional symptoms such as cough, conjunctivitis (red eyes), joint pain, and lymphadenopathy. The combination of fever and generalized rash is widely used as a screening tool during measles outbreaks due to its high sensitivity, albeit with low specificity (39) This low specificity was evident in 2024, where a substantial proportion of suspected cases presenting with fever and rash were not laboratory confirmed, suggesting that these symptoms may have been attributable to other viral infections (42).

Furthermore, symptoms more strongly associated with laboratory-confirmed measles cases were significantly more frequent in 2023 than in 2024. In contrast, symptoms such as rhinorrhea and cough were more commonly reported in 2024, further supporting the likelihood that a proportion of febrile rash illnesses during this period were caused by other circulating viruses, which are known to be prevalent in the study setting (43). Among the clinical features assessed, only joint pain and conjunctivitis were independently associated with laboratory-confirmed measles, consistent with findings from other studies that identify these symptoms as relatively specific indicators of measles infection (40, 44).

It is also important to note that measles cases without rash have been reported in Tanzania and are associated with poor outcomes (45). Following an incubation period of 7–23 days, measles virus infection typically presents with high-grade fever, cough, rhinorrhea, and conjunctivitis lasting 2–3 days, followed by the characteristic maculopapular rash (46). Koplik spots, which are pathognomonic for measles, may appear on the buccal mucosa shortly before rash onset in more than 60% of cases (47). However, the clinical presentation of measles is often nonspecific and may resemble other viral illnesses; typical presentations are particularly common among immunocompromised individuals (39). Following infection, approximately 30% of measles cases develop complications, most commonly pneumonia, otitis media, and diarrhea, with rare occurrences of measles-associated encephalitis. Severe complications are associated with substantial mortality, particularly among infants, where case fatality rates may reach 20–30% (48, 49). A limitation of this study is the lack of data on complications and clinical outcomes, highlighting the need to strengthen surveillance systems to capture these critical variables.

The estimated effectiveness of two doses of measles-containing vaccine (MCV) in preventing laboratory-confirmed measles during the 2023 and 2024 outbreaks was 80.7%. This estimate falls within the wide range reported in studies from the African region, where vaccine effectiveness has been documented to range from 36.5 to 98.4% (50). Notably, 9% of suspected measles cases who had received two doses of MCV were laboratory-confirmed, indicating the occurrence of breakthrough infections. Measles among vaccinated individuals has been reported previously and may be attributed to primary vaccine failure, secondary vaccine failure (waning immunity), or reduced vaccine potency due to cold chain failures (51, 52). The MCV effectiveness estimates are in line with MCV coverage from post-campaign surveys, which have reported coverage ranging from 81.5 to 89% (21, 22).

Consistent with recent findings from a study utilizing quantitative reverse transcription polymerase chain reaction (RT-qPCR) positivity as an outcome, admission status was strongly associated with laboratory-confirmed measles in this analysis (23). Hospital admission is likely to reflect increased disease severity among IgM-positive cases. Furthermore, the majority of admitted patients were either unvaccinated, had unknown vaccination status, or were not yet eligible for vaccination, suggesting a higher risk of severe disease in these groups. This observation aligns with previous studies demonstrating that lack of vaccination is associated not only with increased susceptibility to measles infection but also with more severe clinical outcomes requiring hospitalization (53, 54).

This study has several limitations; (i) data on nutritional status were not available, despite evidence that malnutrition is strongly associated with increased severity of measles virus (MeV) infection (55), (ii) information on complications and clinical outcomes among laboratory-confirmed and admitted measles cases was not collected, limiting the ability to assess disease severity and associated mortality, (iii) a substantial proportion of suspected cases had unknown vaccination status, which may have introduced misclassification bias and affected the analysis of vaccine effectiveness (VE), potentially leading to its underestimation given that the denominator included all suspected cases and (vi) data on doses received through Supplemental Immunization Activities (SIAs) were not captured, as these are typically not recorded on vaccination cards; this may have led to an underestimation of true vaccination coverage.

## 5 Conclusion

Large measles outbreaks during 2023 and 2024 were driven by increasing immunity gaps over time, resulting from the accumulation of under-vaccinated and unvaccinated children in the country. Laboratory-confirmed measles cases were significantly higher across all age categories in 2023, with a noticeable shift in epidemiology toward older age groups and a decline in MCV effectiveness estimates with increasing age. Multiple factors, including partial vaccination and being unvaccinated, were associated with laboratory-confirmed measles cases. A combination of vaccination status, age, and clinical features such as red eyes and joint pain may be used to prioritize and select cases for laboratory confirmation of measles outbreaks in resource-constrained settings. Implementing broader, age- and context-specific catch-up Supplemental Immunization Activities (SIAs) is recommended to close immunity gaps among older children. These strategies should also take into account health system and geographic barriers to vaccination services, as well as the need for sustained caregiver awareness and engagement. Further studies are warranted to investigate immune responses following vaccination, particularly to address concerns raised by stakeholders regarding potential vaccine failure related to cold chain system challenges.

## Data Availability

The raw data supporting the conclusions of this article will be made available by the authors, without undue reservation.

## Glossary

ARU: Attack Rate among the Unvaccinated
ARV: Attack Rate among the Vaccinated
CI: Confidence Interval
CIF: Case Investigation Form
DC: District Council
DIVO: District Immunization and Vaccine Officer
ELISA: Enzyme-Linked Immunosorbent Assay
FGD: Focus Group Discussion
IgM: Immunoglobulin M
KII: Key Informant Interview
MCV: Measles Containing Vaccines
Mev: Measles Virus
MR: Measles Rubella
MSS: Measles Surveillance System
RIVO: Regional Immunization and Vaccine Officer
SOPs Standard Operating Procedures
TDHS: Tanzania Demographic and Health Survey
VE: Vaccine Effectiveness
VPD: Vaccine-preventable disease
WHO: World Health Organization
WUENIC: WHO/UNICEF estimates of national immunization coverage
